# Endovascular Management of Stroke Patients with Large Vessel Occlusion and Minor Stroke Symptoms

**DOI:** 10.7759/cureus.1355

**Published:** 2017-06-15

**Authors:** Saeed A. Alqahtani, Andrew B Stemer, Michael F McCullough, Randy S Bell, Jeffrey Mai, Ai-Hsi Liu, Rocco A Armonda

**Affiliations:** 1 Neurosurgery, Medstar Georgetown University Hospital; 2 Neurology, Medstar Georgetown University Hospital; 3 Neurointerventional Radiology, Medstar Washington Hospital Center; 4 Department of Neurosurgery, Walter Reed Army Medical Center, Washington D.C.

**Keywords:** ischemic stroke, large vessel occlusion, endovascular intervention, mechanical thrombectomy, tpa, outcomes

## Abstract

Endovascular mechanical thrombectomy for stroke patients with large vessel occlusion (LVO) in the anterior circulation has become the standard of care based on several major randomized clinical trials. The successful result reported by these trials constitutes what may be the largest achievement in the history of neurological sciences. However, most of these mechanical thrombectomy trials (except for the multicenter randomized clinical trial of endovascular treatment for acute ischemic stroke in the Netherlands, i.e., MR CLEAN and Extending the Time for Thrombolysis in Emergency Neurological Deficits–Intra-Arterial, i.e., EXTEND-IA) excluded stroke patients with minor to mild stroke symptoms with National Institutes of Health Stroke Scale (NIHSS) scores of six to eight or lower. The median NIHSS score for patients who underwent acute endovascular thrombectomy was approximately 15 to 17 in all trials. To date, the evidence is lacking to support the mechanical thrombectomy in patients with acute stroke and LVO with minor to mild severity on NIHSS score. The purpose of this review was to assess the current data, safety and clinical outcomes in stroke patients with minor to mild symptoms who were treated with endovascular thrombectomy.

## Introduction and background

An untreated large vessel occlusion (LVO) in acute ischemic stroke is associated with poor clinical outcomes. Approximately 80% of patients with an untreated LVO will die within 90 days or become functionally disabled [1]. Treatment with intravenous tissue plasminogen activator (tPA) alone in patients with LVO is associated with a low recanalization rate [2]. For this reason, mechanical thrombectomy along with intravenous tPA has become the standard treatment of acute ischemic stroke patients with LVO and moderate to severe stroke symptoms [3-4]. The recent major clinical trials were mainly focused on patients with moderate to severe stroke symptoms except for the multicenter randomized clinical trial of endovascular treatment for acute ischemic stroke in the Netherlands (MR CLEAN) and Extending the Time for Thrombolysis in Emergency Neurological Deficits–Intra-Arterial (EXTEND-IA) trials [5-9].

There are a significant number of patients with acute ischemic stroke and LVO who can present with mild neurological symptoms. The complex interplay between LVO, blood pressure, collateral status, and clinical symptomatology can result in some patients with LVO presenting with low NIHSS scores. In a large prospective study, higher NIHSS scores had a merely 48% sensitivity for predicting LVO [10]. Most patients present with low NIHSS scores and minimal neurological deficits should not exclude angiographic evaluation in patients with acute stroke symptoms [10-11]. The direct visualization of LVO is extremely important and the treatment modality should include mechanical thrombectomy in appropriate clinical scenarios because the potential recanalization rate with intravenous tPA alone is very low and the chance for neurological recovery is unfavorable.

Based on recent major mechanical thrombectomy trials, the American Heart Association guidelines provided level 1a evidence for mechanical thrombectomy for patients with NIHSS scores of six or more [12]. However, in a large nationwide study, many patients did not receive tPA or early angiographic imaging because of their low NIHSS scores or because, their initial stroke symptoms improved, yet they had poor neurological outcomes [13]. To date, no randomized clinical trials focus on patients with acute ischemic stroke with LVO and low NIHSS scores. The aim of this review is to assess current clinical outcomes and the safety of endovascular mechanical thrombectomies in these patients.

## Review

An acute ischemic stroke with LVO could present with minor stroke symptoms that may result in severe disability or even death if left untreated. Rapidly improving or minor initial stroke symptoms are the most common cause for excluding patients from acute recanalization therapy [14]. One large published study reported 31.2% of patients with acute ischemic stroke did not receive intravenous tPA because of mild or rapidly improving stroke symptoms. Among those with mild strokes or improving symptoms who did not receive therapy, 28.3% were not discharged to home and 28.5% were not able to ambulate and experienced a poor clinical outcome at 90 days [14-18].

Maas, et al. reported that the National Institutes of Health Stroke Scale (NIHSS) scores with > 10 have approximately 81% positive predictive value for large proximal occlusion, but only 48% sensitivity with most of the patients with LVO presenting with lower NIHSS scores were found (55% of patients with NIHSS scores ≤ 10). They concluded that no such NIHSS score threshold could be applied to select a subgroup of stroke patients for acute angiographic imaging without failing to capture most patients with LVO. Failure to visualize the proximal vessel occlusion in acute stroke patients with minor stroke symptoms could have a devastating neurological outcome knowing that the intravenous tPA alone has almost no significant recanalization effect in large proximal occlusion [10]. In these cases, > 60% of the patients will die within 90 days or become functionally disabled [19].

Kim, et al.’s retrospective study of 378 patients with acute ischemic stroke and mild symptoms reported that early neurological deterioration occurred in 55 patients (14.6%) and symptomatic large arterial occlusion was independently associated with neurological deterioration in patients with mild initial stroke presentation [20].

Nedeltchev, et al. studied 162 patients presenting within six hours of acute ischemic stroke onset who did not receive acute thrombolysis therapy because of mild or rapidly improving symptoms. The authors reported 38 patients (23.5%) experienced poor outcomes and 1.3% of patients had died. The presence of proximal LVO drives the chances of poor neurological outcomes to 7.13-fold [18].

Mokin, et al. noted the variability seen in the neurological outcomes of 204 acute ischemic stroke patients with LVO who were admitted with low baseline NIHSS scores (range zero to seven) and excluded from acute intravenous thrombolysis and endovascular intervention. The study reported an increase in disability at the time of discharge for patients with NIHSS scores of zero to two [21].

There is also some evidence to suggest that stroke location may present with falsely mild symptoms (e.g., right-sided infarcts) and poor outcomes without thrombolytic or endovascular treatment. In a study by Cronin, et al, of 66 patients excluded from acute thrombolysis therapy because of rapidly improving or mild symptoms, 16.7% had poor outcomes. Six patients with neurologic deficits had right hemispheric infarcts and one patient also had a cerebellar stroke [22].

The rapidly improving or minor initial deficit is the most common etiology for excluding acute ischemic stroke patients with LVO from thrombolytic therapy. However, the notion of the complex role of leptomeningeal collateral status and good clinical outcomes may affect the decision for mechanical thrombectomy. There is some evidence to support this notion. However, the good collateral status alone is not always optimal and sufficient. As always, recanalization remains critical [23-26].

Miteff, et al. studied 92 acute ischemic stroke patients with LVO and found good collaterals in approximately 55% of patients. The study demonstrated that patients with good collaterals who received an endovascular thrombectomy achieved favorable outcomes. However, in patients with good collaterals but no recanalization, only 38% had good outcomes. Therefore, the presence of good collaterals alone will not prevent unfavorable outcomes and successful recanalization is important [27].

Campbell, et al. evaluated the collateral status and infarction expansion between baseline during the study duration of three to five days using acute magnetic resonance imaging in 88 acute ischemic stroke patients [28]. In 30 patients without reperfusion from day three to five, deterioration in collateral status between baseline and subsequent imaging was strongly associated with infarction volume growth [28].

To date, there is no endovascular randomized clinical trial to address patients with LVO acute ischemic stroke and mild to moderate stroke severity.

Goyal, et al. and HERMES collaborators, in their meta-analysis of the individual patient data from five major mechanical thrombectomy clinical trials, identified a beneficial effect from mechanical thrombectomy for patients across the entire NIHSS [3]. However, only a few patients (177 patients with NIHSS scores ≤ 10) with minor strokes were available for analysis. HERMES collaborators suggested that in clinical practice, the treatment of such patients should be determined based on specific clinical and imaging characteristics, knowing the risk of subsequent neurological deterioration in patients with proximal LVO [3].

Bhogal, et al. studied 41 patients with acute ischemic stroke and LVO with low NIHSS scores (≤ 5) [11]. These patients received mechanical thrombectomy along with standard medical therapy. The successful recanalization was achieved in 87.8% patients (thrombolysis in cerebral infarction [TICI] score ≥ 2b) with only two cases of symptomatic intracranial hemorrhage. At the 90-day follow-up evaluation, 75% of those studied had a modified Rankin Scale (mRS) score from zero to two. There were only three deaths at the 90-day follow-up [11].

Pfaff, et al. performed a retrospective analysis of 484 stroke patients [29]. Thirty-three patients (6.8%) with LVO strokes and NIHSS score of eight or below received acute endovascular thrombectomy with successful recanalization. Twenty-six patients (78.7%) had TICI scores ranging from 2b to three. Only two symptomatic hemorrhage cases were observed. More than 90% of patients had a favorable neurological outcome at 90 days (mRS, 0 to 2: 21 patients, 63.6%l mRS 0 to 3: 30 patients, 90.9%) [29].

Haussen, et al. studied 32 patients with NIHSS scores of ≤ 5 and confirmed LVO via computed tomography angiography. Twenty-two patients (69%) were assigned to thrombolysis therapy and 10 patients (31%) were assigned to the endovascular mechanical thrombectomy [4]. Forty-one percent of patients assigned to the medical therapy had worsening symptoms and required urgent acute thrombectomy despite their median NIHSS score of two. The median time of worsening neurological symptoms was approximately 5.2 hours, consistent with Campbell, et al.’s report on the dynamic and exhaustible nature of leptomeningeal collaterals. The successful reperfusion rate (TICI 2b to three) was achieved in all thrombectomy patients. All of the endovascular thrombectomy group patient’s 90-day mRS ranged from zero to two, but only 77% of patients in the thrombolysis grouped had mRS ranging from zero to two. Three patients died in the medical group, but no deaths were noted in the endovascular intervention group. Haussen, et al. demonstrated a shift towards providing endovascular thrombectomy to patients with a lower NIHSS score and LVO stroke presenting with mild symptoms. Nearly 25% of the medically treated group did not achieve full neurological independence at 90 days [4,28].

Dargazanli, et al. published their study on the impact of acute recanalization therapy on clinical outcomes in patients with low NIHSS scores treated with mechanical thrombectomy [30]. They analyzed 138 patients with acute LVO of the anterior circulation with NIHSS scores < 8 and received acute mechanical thrombectomy. Successful reperfusion (TICI 2b to three) was achieved in 81.2% of patients. At the 90-day follow-up, an excellent outcome (mRS zero to one) was achieved in 69 patients (65.0%), and favorable outcomes (mRS ≤ 2) were achieved in 108 patients (78.3%). The death occurred in seven patients (5.1%). These findings are consistent with the previously mentioned studies. The probability of excellent outcomes increased with recanalization grades with a rate of 34.6% in patients with failed or poor recanalization, 61.7% in patients with TICI 2b reperfusion, and 78.5% in patients with TICI three reperfusion [30].

This review article has several limitations. The studies mentioned here are retrospective, observational in nature, or self-reported safety outcomes lacking central adjudication of imaging or neurological outcomes, without control group comparators except for Haussen, et al. and his intention-to-treat analysis. The small sample size of the patients treated in these studies does not allow for the recommendation of a minimum NIHSS cutoff for acute mechanical thrombectomy.

Although no randomized clinical controlled trial data are available, cumulative clinical evidence supports treating patients presenting with minor to mild stroke symptoms and LVO with timely endovascular thrombectomy.

## Conclusions

Acute endovascular mechanical thrombectomy in patients presenting with minor to mild stroke symptoms and proximal large vessel occlusion (LVO) appears to be favorable and safe and carries a high chance of successful recanalization with good clinical outcomes. However, future randomized controlled clinical trials are necessary for further evaluation of the benefits of endovascular mechanical thrombectomy compared with the best medical therapy in these patients.
